# Mapping the Behavioral Weight Management Ecosystem in the East of England to Inform the Implementation of Electronic Signposting

**DOI:** 10.1002/osp4.70155

**Published:** 2026-06-05

**Authors:** Natalie An Qi Tham, Fredrik Kjell Bodell, Helen M. Parretti, Helena Jopling, Zarnie Khadjesari

**Affiliations:** ^1^ School of Health Sciences University of East Anglia Norwich UK; ^2^ Faculty of Industrial Design Engineering Delft University of Technology Delft the Netherlands; ^3^ Norwich Medical School University of East Anglia Norwich UK; ^4^ Department of Public Health West Suffolk NHS Foundation Trust Bury St Edmunds UK

**Keywords:** ecosystem mapping, obesity, weight management

## Abstract

**Background:**

Obesity is a leading cause of ill health in England and places a substantial burden on health systems and the economy. Behavioral weight management services (WMS) are central to reducing obesity‐related risk, but their reach remains limited. Electronic signposting (eSignposting), which uses electronic health records and digital communication to connect patients with appropriate services, may improve access to behavioral WMS. Effective and equitable implementation of eSignposting requires a comprehensive understanding of the behavioral WMS ecosystem, including its key components and interdependencies.

**Objective:**

This study aimed to characterize the behavioral WMS ecosystem in the East of England (Norfolk, Suffolk, and North‐East Essex) to identify opportunities and considerations for implementing eSignposting.

**Methods:**

A qualitative study was conducted using semi‐structured interviews with professional stakeholders (*n* = 11) involved in the commissioning, referral, and delivery of behavioral WMS. Directed content analysis was used to develop ecosystem maps and a comprehensive inventory of services across the region.

**Results:**

The findings revealed a complex behavioral WMS landscape spanning the local authority, national, commercial, and voluntary sectors. Self‐referral and primary care referral emerged as the predominant routes to accessing behavioral WMS. Ecosystem maps identified key entry points where eSignposting could maximize reach and impact. Analysis of inter‐stakeholder relationships also highlighted potential unintended consequences of eSignposting, including increased pressure on local services, preferential uptake of digitally accessible commercial programmes, and the risk of digital exclusion among underserved populations, enabling these issues to be proactively addressed in the future design and implementation of eSignposting.

**Conclusion:**

The behavioral WMS ecosystem in East England is multifaceted and interconnected. Ecosystem mapping provides valuable insight into referral pathways and stakeholder relationships, supporting the development of effective and equitable eSignposting strategies to improve access to behavioral WMS while minimizing risks to service equity and sustainability.

**Abbreviations**
BMI: body mass index
GP: general practitioner
HCP: healthcare professional
ICB: integrated care board
NHS: National Health Service
UK: United Kingdom
VCSE: voluntary, community, social, and enterprise
WMS: weight management services

## 1 Introduction

Overweight and obesity are among the leading public health challenges in England, affecting over 60% of adults [1]. The health, social, and economic consequences are considerable: obesity increases the risk of conditions such as type 2 diabetes, cardiovascular disease and musculoskeletal disorders, and is estimated to cost the National Health Service (NHS) £6.5 billion annually [1, 2]. An estimated 42 million people in the UK could be living with overweight or obesity by 2040, representing approximately 71% of the population [3]. The disparity between the obesity levels of the most and least deprived sections of the population could also increase by more than half by 2040, meaning that the worst impacts of obesity and overweight may be felt by those in the most deprived communities [3]. There is therefore an urgent need to prioritize interventions that reduce the burden of overweight and obesity, particularly for those who need them the most.

In England, the management of overweight and obesity is delivered across a structured, tiered system of care. Universal services, which are also referred to as Tier 1 services, focus on prevention and deliver early intervention for the general population. These services involve public health campaigns and brief interventions that emphasize healthy lifestyle principles for weight management. Behavioral weight management services (WMS), often referred to as Tier 2 services, comprise structured programmes that incorporate evidence‐based approaches to changing and managing diet, physical activity, and lifestyle. Specialist services, referred to as Tier 3 and 4 services, involve specialist care delivered by multidisciplinary teams trained in nutritional counseling, psychological and pharmacological therapies, as well as bariatric surgery [4]. Integrated care boards (ICBs) are typically responsible for planning and funding specialist WMS, whereas the commissioning of universal and behavioral WMS usually falls under Local Authorities, with some joint commissioning arrangements in place [5].

Within this context, behavioral WMS represents a frontline intervention. These programmes are typically delivered over 12 weeks and are available to adults with a body mass index (BMI) ≥ 30 kg/m^2^, with thresholds lowered (BMI ≥ 27.5 kg/m^2^) for individuals at increased risk of chronic weight‐related conditions, such as those from ethnic minority groups [6]. In 2021, the UK government invested £100 million to extend support for weight management to children, adults, and families, of which £30.5 million was allocated specifically to expanding the delivery of behavioral WMS through the 2021/2022 Adult Weight Management Services Grant [7]. One outcome of this investment was the NHS Digital Weight Management program, a free 12‐week intervention offering personalized weight management support through online content such as evidence‐based digital resources, progress tracking, and remote coaching [8]. Behavioral WMS has demonstrated clinically meaningful and sustained weight loss in both trial and real‐world settings [9].

Despite the availability of these effective services, the reach of behavioral WMS remains limited. Analyses of primary care records have found historically low referral rates: among individuals with a recorded diagnosis of overweight or obesity, only around 5% of men and 4% of women were referred to any WMS between 2005 and 2012 [10]. More recently, studies analyzing nearly two million primary care records in England from 2007 to 2020 found that only 3% of patients with overweight or obesity received a referral to any WMS, with even lower rates of referral to behavioral WMS [11]. Initiatives such as the National Enhanced Service for weight management, which was introduced in 2021 to incentivize referrals by reimbursing GP (general practitioner) practices for each eligible referral made, have not led to measurable improvements in referral activity [12].

It is worth noting that in other healthcare contexts, such as in the United States, programs equivalent to behavioral WMS are commonly referred to as lifestyle modification interventions, and the primary challenge is one of utilization rather than that of reach [13]. In England, however, the challenge is twofold: low rates of referral mean that many eligible patients do not enter the weight management pathway, while those who do are often lost to enrollment or engagement at subsequent stages. For example, between 2021 and 2022, only 65% of individuals referred to a behavioral WMS enrolled, with particularly low participation rates among underserved populations: just 15% of enrollees were from ethnic minority groups and 26% from the most deprived areas in England [14], although it should be noted that this period coincides with the aftermath of the COVID‐19 pandemic, which may have affected referral and enrollment patterns due to disruptions in healthcare service delivery [15]. These gaps in access and engagement contribute to persistent inequities in obesity outcomes and raise uncertainty about the long‐term clinical and cost‐effectiveness of behavioral WMS [16, 17]. For example, an evaluation of seven behavioral WMS in the North of England found that only two met the National Institute of Health and Care Excellence's benchmark of 30% participants achieving ≥ 5% weight loss at 12 weeks [17].

Several factors may limit the reach of behavioral WMS. Uncertainty among healthcare professionals (HCPs) about the availability of services and the appropriate pathways through which patients should be referred to impede timely referrals [18]. This is compounded by fragmented and inconsistent regional provision of services due to differing commissioning models, program structures, delivery methods, referral protocols, and eligibility criteria [18]. Additional barriers reported by HCPs include limited consultation time, insufficient training in obesity care, a perceived lack of suitable services, discomfort initiating conversations about weight during consultations, and concerns about jeopardizing patient rapport [19, 20]. Patients similarly report limited awareness of available services, unclear eligibility and referral processes, and fear of stigma as barriers to engagement [18]. Consequently, obesity often goes unaddressed in primary care for many years after onset [19].

To address these challenges, new approaches are needed to increase the reach, accessibility, and equity of behavioral WMS. One promising solution is electronic signposting (eSignposting), which involves the use of digital communication (e.g., text messages) to proactively connect eligible patients identified through health record data with appropriate services [21]. While digital and remote delivery models for weight management—including text messaging and phone or video‐based health coaching—have been widely adopted internationally [22], their use for proactive patient signposting to behavioral WMS is a more recent development, particularly in England [21]. eSignposting can be viewed as an adjunctive intervention [23], whose purpose is to increase the uptake of effective behavior change interventions. For example, one study found that 14% of patients who self‐identified as smoking or consuming alcohol at risky levels interacted with behavior change applications after receiving a targeted text message recommending their use [21]. Such interventions have also proven effective in improving engagement with tobacco cessation [24] and cancer screening programs [25] need for clinician‐initiated steps within the referral pathway, eSignposting offers a scalable way to streamline patient access to behavior change interventions and reach populations less likely to be engaged through traditional methods.

However, little is known about how best to embed eSignposting within the complex obesity management ecosystem. Obesity management involves a diverse set of stakeholders, including service providers, HCPs, commissioners, and digital infrastructure teams whose interactions shape whether, when, and how patients are referred to services, as well as how those services are delivered and commissioned [5, 18]. Without a clear understanding of this variation and the stakeholders involved, efforts to implement innovations like eSignposting risk being fragmented, poorly targeted, or unsustainable [26]. This is because effective implementation of healthcare innovations requires alignment and fit with existing organizational workflows (e.g., referral pathways), professional roles (e.g., HCPs who act as referrers), and service structures (e.g., how behavioral WMS are organized, commissioned, and delivered) within a particular healthcare system [27].

Developing an accurate and comprehensive understanding of how the behavioral WMS operates in practice is key to implementing innovations such as eSignposting. This often involves the development of visual tools, such as *ecosystem maps*, that represent the key components of a healthcare ecosystem and the relationships between them [28]. These diagrammatic representations visualize the structural and institutional landscape in which innovations are embedded by incorporating input from stakeholders within the system [28]. In the context of eSignposting, they can clarify which behavioral WMS are available for patients to be signposted to, identify specific referral and commissioning pathways that should be leveraged to facilitate its implementation, and highlight the stakeholders (e.g., commissioners, HCPs, service providers, digital infrastructure teams) that need to be engaged. As such, ecosystem maps are essential for supporting the strategic integration of eSignposting into local obesity care pathways.

This study aimed to characterize and map the behavioral WMS ecosystem in the East of England, with a view to offering actionable insights to inform the implementation of eSignposting. The behavioral WMS ecosystem is understood here as the interconnected network of services, stakeholders, referral mechanisms, and commissioning structures that collectively enable access to and delivery of behavioral WMS. This study is part of a broader programme of research (eSign project), which is working with NHS hospital trusts across the East of England to implement eSignposting, with the aim of improving patient access to and engagement with behavioral WMS. To date, this work includes a weight management needs assessment in under‐resourced communities elicited using storyboarding and a realist lens [29] and a realist review of eSignposting to interventions that prevent cancer [30].

## 2 Methods

### 2.1 Design

A qualitative study using ecosystem mapping methodology [28] was conducted to characterize the behavioral WMS landscape across Norfolk, Suffolk, and North‐East Essex—a geographically bounded region selected to reflect the distinct commissioning and service delivery arrangements operating within this area. Data collection and analysis took place concurrently in a flexible, iterative process. Interview data were examined through categorical aggregation, where recurring patterns and shared stakeholder accounts were identified to develop a holistic understanding of the ecosystem. The findings were used to construct and refine the ecosystem maps, which were iteratively refined in response to participant feedback.

### 2.2 Participants and Recruitment

Participants were purposively sampled professionals actively engaged (within the past 2 years) in the commissioning, referral, or delivery of behavioral WMS across Norfolk, Suffolk, and North‐East Essex. These areas fall within the footprints of two of several ICBs operating in the East of England region, namely, Norfolk and Waveney ICB, and Suffolk and North‐East Essex ICB. While ICBs do not directly commission behavioral WMS, they play a strategic role in shaping local service ecosystems and interfacing with public health commissioning led by Local Authorities [5].

Eligible individuals within the research team's existing professional network were invited via email, and additional participants were identified through snowball sampling based on recommendations from initial contacts. This recruitment approach resulted in a 100% response rate. All participants provided written informed consent prior to the interview.

### 2.3 Data Collection

Data were collected through semi‐structured online interviews conducted via Microsoft Teams. A flexible interview schedule guided in‐depth exploration of topics including available WMS, eligibility criteria, service details, referral pathways, and commissioning mechanisms. Interviews were conducted by a research associate with prior qualitative research experience (NAQT). All but one interviews were conducted individually; in one instance, two participants at the same organization were interviewed together at their request. Interviews lasted between 18 and 46 min, were audio‐recorded and transcribed verbatim.

The sample size was guided by the concept of information power, which determines sample adequacy based on the richness and relevance of the data rather than its quantity [31].

### 2.4 Data Analysis

Transcripts were imported into NVivo software for directed content analysis [32]. Data were coded according to predefined categories relevant to the study aim, including components of the behavioral WMS ecosystem (e.g., services, stakeholders) and their interrelationships (e.g., referral and commissioning pathways). Text segments describing service features, pathways, or relationships between stakeholders were coded at the phrase level. Coded data were used to construct ecosystem maps in Lucidchart [33], an online platform selected for its capacity to visually represent complex inter‐stakeholder relationships, as well as for being low‐cost and accessible. Separate maps were developed for each county to account for regional variation.

In parallel, a comprehensive inventory of identified behavioral WMS was compiled by combining interview data with targeted online searches of the available websites of individual services to verify and supplement the information obtained from interviews. The inventory was developed and maintained in Microsoft Excel and provides a list of the identified behavioral WMS and their aims, eligibility criteria, delivery format (e.g., mode, duration, frequency), referral and commissioning mechanisms, and cost to participants. All coding and construction of the ecosystem maps and service inventory were completed by NAQT. This study received ethical approval from the Research Ethics Committee at the Faculty of Medicine and Health Sciences, University of East Anglia (approval number ETH2425‐1723).

## 3 Results

### 3.1 Participant Characteristics

A total of 11 professional stakeholders took part in the study at least once. Participants included GPs (*n* = 1), clinical leads at hospital trusts (*n* = 1), behavioral WMS providers (*n* = 3), health innovation partners supporting the development and implementation of services (*n* = 1), and representatives from Local Authorities (*n* = 3) and ICBs (*n* = 2). After 11 interviews, the study was judged to have collected sufficient information to address its aim, with participants' perspectives showing convergence and key aspects of the behavioral WMS ecosystem well represented. Draft ecosystem maps and the service inventory were circulated to participants for review via email. Feedback was received from *n* = 5 participants (response rate = 45%) and was iteratively incorporated into the ecosystem maps and service inventory to ensure they were accurate and comprehensive.

### 3.2 Service Landscape

The analysis demonstrated a structured yet multifaceted behavioral WMS landscape within East England. A total of *n* = 11 behavioral WMS were identified, with core provision in each county centered around programs funded by Local Authorities. In Suffolk, the primary locally commissioned service (Feel Good Suffolk) was delivered in‐house, being both funded and directly provided by the Local Authority. In contrast, the locally funded services in Norfolk and North‐East Essex (Your Health Norfolk and Essex Wellbeing Service) were commissioned by the Local Authority but delivered by external partners, most commonly community interest companies that reinvest profits into improving public health, such as by providing weight management support within the community.

Commercial organizations such as Slimming World, WW (formerly Weight Watchers), and the Noom digital behavior change application were also identified as prominent WMS providers in the region. Their inclusion in referral pathways was an indication of their extensive reach within the community, sometimes as commissioned services (as in Norfolk) and other times as self‐funded private schemes (as in Suffolk and North‐East Essex). For clarity and due to significant differences in program formats and eligibility criteria, the commissioned and non‐commissioned Slimming World schemes were documented as separate services in this study. The provision of voluntary, community, and social enterprise (VCSE) services was also evident but less widespread across the region. The social enterprise Man v Fat Football was active in all three counties, offering a weight management program specifically targeting males that emphasized regular physical activity through peer‐supported football leagues.

In all counties, local programmes operated in parallel with national initiatives such as the NHS Digital Weight Management Program, the Type 2 Diabetes Path to Remission Program, and the Healthier You: Diabetes Prevention Program. These national services provided additional entry points for specific populations at a greater risk of developing obesity‐related complications, such as patients with hypertension or Type 2 diabetes.

### 3.3 Service Delivery

The vast majority of the identified services (*n* = 9 of 11 services, 82%) provided in‐person support for weight management, although the extent of face‐to‐face provision varied. For example, the Local Authority‐commissioned program in Norfolk operated exclusively as an in‐person service, delivered via group sessions held in community venues. In contrast, the Local Authority‐commissioned program in Essex primarily delivered remote support via scheduled telephone consultations with trained advisors, supplemented with drop‐in sessions that participants could attend in‐person if required. Most services (*n* = 8 of 11, 73%) offered some form of remote support, including self‐paced access to recorded sessions available online, or consultations with advisors via video conferencing or telephone. Program materials were also typically available to participants via email or digital mobile applications. Over half of the services (*n* = 6 of 11, 55%) operated hybrid models, offering both in‐person and remote access options. This provision of multi‐modal forms of support aligns with current best practices that emphasize the accessible patient‐centered modalities of care designed to integrate into daily lives [4]. Only two (18%) services operated entirely remotely, delivering program material primarily via online websites and digital applications.

Nationally commissioned programmes were typically subcontracted to private providers such as Xyla, Counterweight, or Oviva. These organizations deliver the programmes on behalf of the NHS, providing structured forms of behavioral intervention for weight management using digital platforms, remote coaching, and, in some cases, face‐to‐face support.

Service durations ranged from 10 weeks to 12 months, with most services delivering a 12‐week program (*n* = 4 of 11, 36%). Four services (36%) operated on a rolling membership model, whereby participants were able to use the service as long as they had an active subscription. In order to access these services, participants had to pay out‐of‐pocket and did not receive any public health or NHS funds. On the contrary, services that were commissioned by Local Authorities (*n* = 4 of 11, 36%) or the NHS (*n* = 3 of 11, 27%) were free to access. The frequency of the services also varied, with most programmes delivering weekly sessions (*n* = 6 of 11, 54%) and some following structured non‐weekly formats (*n* = 5 of 11, 45%). Non‐weekly formats typically consisted of either a fixed number of sessions delivered over the program duration or self‐paced, remotely accessible materials, such as app‐based content that participants could use at their convenience.

### 3.4 Program Content

The services varied considerably in content, although most focused on supporting sustainable behavior change through a combination of nutritional advice, physical activity promotion, and behavior change techniques. For example, locally commissioned services in Norfolk and Suffolk and the nationally commissioned NHS Digital Weight Management Program delivered evidence‐based support on diet, exercise, sleep, and stress management. Approaches to encouraging physical activity varied, with some services (e.g., Man v Fat Football) centered around structured, group‐based exercise sessions and others (e.g., Slimming World) focusing on physical activity education and self‐directed exercise.

The commercial providers offered proprietary programmes that used specific strategies to help participants adopt and sustain healthier habits to manage their weight. For example, Slimming World advocated a flexible eating approach that encourages members to eat nutritionally balanced meals without counting their calories while gradually increasing their physical activity. Similarly, WW (Weight Watchers) encouraged healthier food choices using a points system, assigning point‐values to foods based on their nutritional profile and giving members a personalized points allowance to guide portion control and healthy substitutions. Both programmes combined in‐person group sessions with digital tools such as app‐based meal planners and activity trackers, as well as remote sessions for those unable to attend in person. In contrast, the Noom program was delivered entirely through a mobile application that offered digital‐only weight management support. This application allowed users to track their meals, access bite‐sized interactive lessons (e.g., on strategies to manage stress and weight), receive remote coaching on how to adopt, self‐monitor, and maintain healthier habits, and participate in peer support groups designed to foster a sense of social connection and shared accountability among users working toward similar goals.

Some programmes targeted specific populations or those with obesity‐related comorbidities. For example, Man v Fat Football engaged men through organized group football leagues that reward both on‐pitch performance and progress toward healthier behavior change goals. At the national level, the Healthier You: Diabetes Prevention Program and the Type 2 Diabetes Path to Remission Program provided structured support in managing diabetes. Specifically, the Healthier You program promoted long‐term dietary and physical activity changes to improve blood glucose control and prevent diabetes, while the Path to Remission program involved an intensive low‐energy diet aimed at achieving remission from Type 2 diabetes.

### 3.5 Eligibility Criteria

Although the identified services specified different eligibility criteria, many followed similar baseline requirements. The majority of the services (*n* = 10 of 11, 91%) were inclusive, with only one being accessible only to men. Most services (*n* = 10 of 11, 91%) required that individuals be at least 18 years old to participate, though one service accepted participants from the age of 16 years. An upper age limit was only applied by two services (18%) and ranged from 65 to 80 years, although this could be overridden at clinical discretion. For example, in the Healthier You program, individuals above age 80 could access the service if referred by a GP. The minimum BMI criteria for participation ranged from 25 kg/m^2^ to 30 kg/m^2^, with ≥ 30 kg/m^2^ being the most common (specified by *n* = 4 of 11 services, 36%). Where the minimum BMI criterion was set at ≥ 30 kg/m^2^ for participants of a White ethnic background, this threshold was typically lowered for individuals from minority ethnic groups, in line with national recommendations on ethnicity‐based risk stratification [6]. Only one of the nationally commissioned services, the Healthier You program, did not use BMI as an exclusion criterion and instead enrolled participants based on their risk of developing Type 2 diabetes. This reflects its primary focus on diabetes prevention rather than weight management.

Residency within the relevant county was a condition of participation for locally commissioned services, while some nationally commissioned services required the presence of obesity‐related comorbid conditions such as diabetes or hypertension. Common exclusion criteria included being pregnant, having an active eating disorder, having recently undergone bariatric surgery, or having a medical or mental health condition that could impede engagement with the program. Some Local Authority‐commissioned services excluded individuals who had recently accessed the service to prioritize access for new participants.

### 3.6 Referral Pathways

Across all three counties, self‐referral was found to be the main method of accessing a behavioral WMS, being applied by most services (*n* = 8 of 11, 73%). Self‐referral typically requires that patients complete a referral form available on the service website. Primary care referral was the second most common pathway into a service (*n* = 5 of 11, 46%), where patients had to be referred to a service by their healthcare provider such as a GP, pharmacist, practice nurse, social prescribing link worker, or other primary care practitioner such as a dietitian.

Some programs, such as the NHS Digital Weight Management program, accepted referrals exclusively from primary care. In contrast, services that were commissioned by Local Authorities generally adopted broader referral frameworks, accepting referrals not only from primary care but also from secondary care and other healthcare professionals with access to patient records, including hospital‐based physiotherapists and diabetes teams. An exception was Your Health Norfolk, which only accepted patient self‐referrals via its website. Similarly, commercial and VCSE programs required that participants self‐enroll via their website on a paid membership basis, with no involvement from HCPs.

### 3.7 Commissioning Structures

The behavioral WMS in all three counties used similar commissioning structures and received funding from Local Authorities and NHS England. Local Authorities are not under statutory obligation to provide behavioral WMS [34]; however, they remain the primary commissioners. This was illustrated by the primary program in each county, which was both funded and overseen by their respective Local Authorities.

National commissioning delivered additional services in this landscape, with NHS England commissioning several programs targeted at individuals with specific obesity‐related comorbidities such as Type 2 diabetes and hypertension. These centrally commissioned services not only enhance the overall capacity of the system to offer weight management support and address the needs of at‐risk groups but also broaden access for individuals who may be unable to participate in programmes limited to in‐person delivery via digital WMS provision (e.g., the NHS Digital Weight Management Program).

VCSEs and commercial organizations generally operated under patient self‐pay models, though there was some variation between regions in terms of commissioning support. For example, Slimming World receives Local Authority funding in Norfolk but not in Suffolk or North‐East Essex. Similarly, while Man v Fat football is not funded by public health in the East of England, it has historically received Local Authority funding in regions that were not part of this study, such as in Nottinghamshire [35].

### 3.8 Ecosystem Maps

**FIGURE 1:**
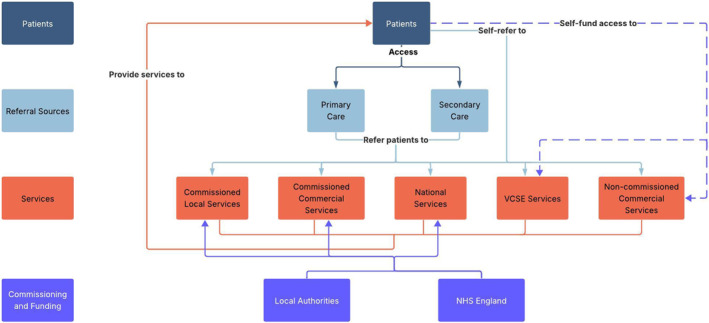
Map of the behavioral weight management ecosystem in East England, depicting key services, stakeholders, and their interrelationships. VCSE = voluntary, community, social, and enterprise.

**FIGURE 2:**
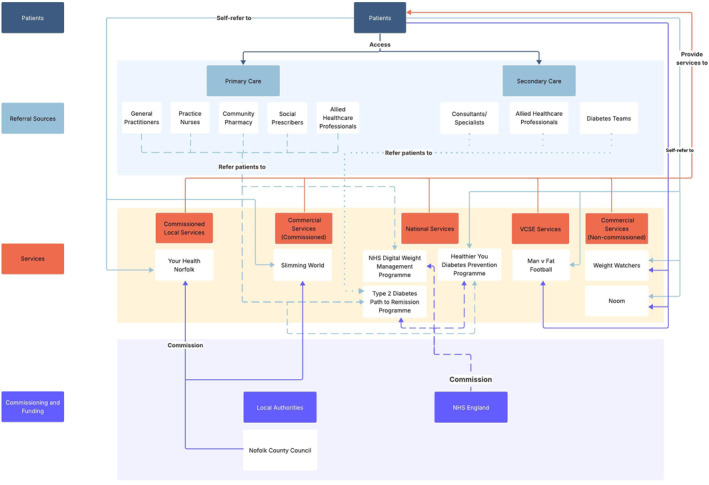
Map of the behavioral weight management ecosystem in Norfolk. VCSE = voluntary, community, social, and enterprise.

**FIGURE 3:**
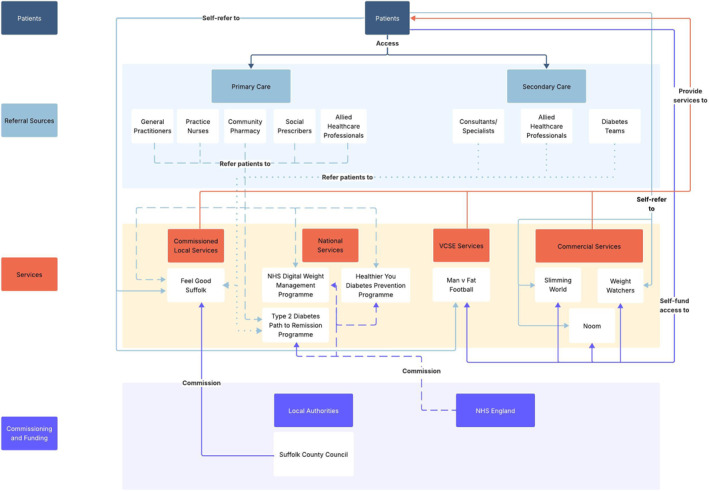
Map of the behavioral weight management ecosystem in Suffolk. VCSE = voluntary, community, social, and enterprise.

**FIGURE 4:**
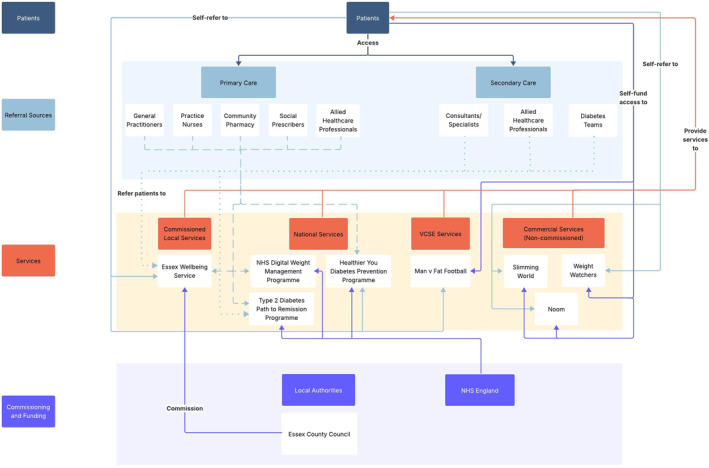
Map of the behavioral weight management ecosystem in North‐East Essex. VCSE = voluntary, community, social, and enterprise.

Figure 1 illustrates the core structure of the regional behavioral WMS ecosystem, highlighting its key components and their relationships. Figures 2, 3, 4 display the county‐specific ecosystem maps for each of Norfolk, Suffolk, and North‐East Essex. Dynamic, interactive versions of these maps are available through an online Lucidchart link: Ecosystem maps. Table 1 outlines a detailed inventory of behavioral WMS services available across the region.

**TABLE 1:** Behavioral weight management services in Norfolk, Suffolk, and North‐East Essex.

Service	County	Details on service	Eligibility criteria	Mode of delivery	Duration	Referral route	Frequency	Delivery format	Cost to participants
Your health Norfolk	Norfolk	Program designed to support participants in achieving a healthy weight and improving overall wellbeing through evidence‐based guidance on healthy eating, physical activity, sleep, and sustained habit formation. Participants can choose between a nutrition‐only course or a combined nutrition and exercise program.	Inclusion: Age ≥ 18 BMI ≥ 30 kg/m^2^ for White Caucasian individuals or ≥ 27.5 kg/m^2^ for individuals from ethnic minority backgrounds or those with comorbidities Resident in Norfolk Exclusion: Currently pregnant Participation in a funded WMS within the past 12 months Paid membership with commissioned Slimming world or Your health Norfolk within the past 3 months	In person	10 weeks	Self‐referral	Weekly	Group	Free
Feel good Suffolk	Suffolk	Program designed to support sustainable lifestyle changes for weight management through nutrition education, behavior change techniques, and self‐management skills, including stress and sleep management.	Inclusion: Age ≥ 18 Motivated to achieve a healthy weight BMI ≥ 30 kg/m^2^ for White Caucasian individuals or ≥ 27.5 kg/m^2^ for individuals from ethnic minority backgrounds or those with comorbidities Resident in Suffolk Individuals with BMI ≥ 40 kg/m^2^ must speak with their GP or a qualified healthcare professional before submitting a self‐referral.	In person and remote (online self‐paced access to recorded sessions)	12 weeks	Healthcare professional (primary and secondary care) referral and self‐referral	Weekly	Group	Free
Essex wellbeing service	North‐East Essex	Two main programmes are offered: My weight Matters: An evidence‐based program providing guidance on healthy eating and physical activity, while supporting participants in developing sustainable, long‐term lifestyle changes. The low Carb program: A self‐guided program offering information, skills, tips, and support to help participants adopt a low‐carbohydrate approach to achieving their health goals.	Inclusion: Age ≥ 18 BMI ≥ 25 kg/m^2^ Resident in Essex Exclusion: Has a current or historical diagnosis of an eating disorder	In person and remote (telephone consultation and self‐paced access to program material via email and the AmaraHealth digital app)	12 weeks	Healthcare professional (primary and secondary care) referral and self‐referral	Three telephone sessions over 12 weeks	Individual	Free
Slimming world (commissioned by Norfolk county council)	Norfolk	Program designed to support sustained lifestyle changes for weight management through consumption of healthy, everyday foods without calorie counting. This is supported by a tailored physical activity component that promotes gradual increases in activity at participants' own pace.	Inclusion: Age ≥ 18 BMI ≥ 30 for White Caucasian individuals or ≥ 27.5 for individuals from ethnic minority backgrounds or those with comorbidities Resident in Norfolk Exclusion: Currently pregnant Participation in a funded WMS within the past 12 months Paid membership with commissioned Slimming world or Your health Norfolk within the past 3 months	In person	12 weeks	Self‐referral	Weekly	Group	Free
Slimming world	Suffolk and North‐East Essex	Commercial program designed to support sustained lifestyle changes for weight management through an emphasis on consuming healthy, everyday foods without calorie counting. This is supported by a tailored physical activity component that promotes gradual increases in activity at participants' own pace.	Inclusion: Age ≥ 16	In person and remote (online virtual workshops and self‐paced access to program material via the Slimming world app)	Rolling membership	Self‐referral	Weekly	In‐person group sessions, with individual remote access to digital program material	£5 joining fee. Weekly fee of £5.95 (reduced rates for 16–17‐year‐olds and over‐60 s). Online membership starts at £60 for 3 months, with higher‐tier digital packages available.
WW (weight watchers)	Norfolk, Suffolk, and North‐East Essex	Commercial program designed to support weight management through sustained changes to diet and behavior. A points system assigns values to foods based on calories, sugar, saturated fat and protein. Members receive a personalized daily points allowance and are encouraged to eat nutrient‐dense “ZeroPoint” foods that do not require tracking.	Inclusion: Age ≥ 18 Exclusion: Currently pregnant	In person and remote (online virtual workshops and self‐paced access to program material via the WW (weight Watchers) app)	Rolling membership	Self‐referral	Weekly	In‐person group sessions, with individual remote access to digital program material	Monthly fee of £5.65 (1‐month subscription), £10 (6‐month subscription), or £18.95 (12‐month subscription).
Noom	Norfolk, Suffolk, and North‐East Essex	Commercial app‐based weight management program that combines calorie tracking with behavior change strategies, daily educational content, and remote health coaching to support sustainable lifestyle changes.	Inclusion: Age ≥ 18 Has access to a smartphone	Remote (self‐paced access to program material via the Noom digital app)	Rolling membership	Self‐referral	Daily use of Noom digital app	Individual	Typically £22/month, but costs vary depending on promotions and length of commitment
Man v Fat football	Norfolk, Suffolk, and North‐East Essex	Football league for men looking to improve their health and fitness. Participants earn points for both team performance and personal progress toward healthier lifestyle goals, creating a supportive, goal‐oriented environment.	Inclusion: Age ≥ 18 Male BMI ≥ 27.5 kg/m^2^	In person	Rolling membership	Self‐referral	Weekly	Group	£9.99 registration fee. Monthly membership fee ranging from £31 to £33.50.
NHS digital weight management program	Nationwide	An online behavioral and lifestyle program providing personalized support via digital tools (apps, online resources, and coaching) to promote healthier eating, increased physical activity, and sustainable behavior change. Accessible via smartphone or computer with internet access.	Inclusion: Age 18–80 BMI ≥ 30 kg/m^2^ for White Caucasian individuals or ≥ 27.5 kg/m^2^ for individuals from ethnic minority backgrounds or those with comorbidities Has a diagnosis of diabetes, hypertension, or both Has access to a smartphone, tablet, or computer with internet access Exclusion: Currently pregnant Has moderate or severe frailty Has an active eating disorder Has had bariatric surgery within the past 2 years Those for whom a weight management program poses greater harm than benefit (i.e., age ≥ 80)	Remote (self‐paced access to program material via the Xyla digital app). Remote telephone/video consultation may be offered to those with lower likelihood of completing the program (e.g., individuals who are younger, male, from ethnic minority backgrounds, or living in more deprived areas).	12 weeks	Healthcare professional (primary care)	Weekly modules with daily self‐monitoring via digital app	Individual	Free
NHS type 2 diabetes Path to remission program	Nationwide	Program designed to improve blood glucose control, reduce diabetes‐related medication, and potentially achieve remission of type 2 diabetes by combining a low‐calorie diet (800–900 calories/day via soups and shakes) with structured behavior change support to gradually reintroduce healthy meals.	Inclusion: Age 18–65 BMI ≥ 27 kg/m^2^ for White Caucasian individuals or ≥ 25 kg/m^2^ for individuals from ethnic minority backgrounds or those with comorbidities Has had a diagnosis of type 2 diabetes within the past 6 years Exclusion: Currently pregnant Has moderate or severe frailty Has an active eating disorder Has had bariatric surgery within the past 2 years Those for whom a weight management program poses greater harm than benefit (e.g., age ≥ 80)	Choice of in person or remote (telephone consultation and self‐paced access to program material via a digital app, operated by Oviva in Norfolk; telephone/video consultation and self‐paced access to program material via a digital app, operated by Counterweight in Suffolk and North‐East Essex)	12 weeks of total diet replacement alongside 12 months of monitoring and support (comprising 4 weeks of food introduction and 9 months of behavior change support)	Healthcare professional (primary care and secondary care)	Total diet replacement treatment is continuous for the first 12 weeks; frequency of monitoring and support depends on the provider (Oviva or Counterweight)	Individual	Free
Healthier you: Diabetes prevention program	Nationwide	Evidence‐based lifestyle change program for individuals at high risk of developing type 2 diabetes. Comprises personalized diet and physical activity guidance, as well as a digital option that includes wearable activity tracking, access to health coaching via an app, and online peer support to promote sustainable behavior change.	Inclusion: Age ≥ 18 Has non‐diabetic hyperglycemia identified by blood test within the past 12 months Has HbA1c of 42–47.9 mmol/mol Has fasting plasma glucose of 5.5–6.9 mmol/L Exclusion: Currently pregnant Has blood results suggesting type 2 diabetes Age ≥ 80 without prior consideration for the risks and benefits of a program Has had bariatric surgery within the past 2 years Has an active eating disorder	Choice of in person or remote (telephone consultation and self‐paced access to program material via a digital application operated by Xyla)	9 months	Healthcare professional (primary care), with self‐referral accepted from individuals with a history of gestational diabetes mellitus	Six fortnightly and 7 monthly sessions	Choice of in‐person group sessions or individual remote access to digital program material	Free

Abbreviations: BMI = body mass index, HbA1c = hemoglobin A1c.

## 4 Discussion

This study provides a comprehensive overview of the behavioral WMS landscape across the East of England, with a view to guiding the implementation of a novel service innovation, eSignposting, to promote overweight and obesity management at the population level. The ecosystem maps outline the essential components of the regional behavioral WMS ecosystem, illuminating its constituent stakeholders, their interrelationships, and the range of WMS that are available via these networks.

Our findings are consistent with previous research describing the complexity of behavioral overweight and obesity management services across England [34], pointing to a diverse regional ecosystem encompassing local, national, commercial, and community‐based provision. Most Local Authority‐commissioned services prioritized accessibility, offering free programs delivered through hybrid models that combined in‐person and remote participation. Nationally commissioned services were also designed to be accessible, while often targeting individuals at higher risk due to obesity‐related comorbidities. These flexible delivery models directly address known barriers to engagement with WMS, such as fixed program timings and venues [36]. They also align with national recommendations calling for more inclusive and personalized approaches to obesity management, with a stronger focus on ensuring equitable access for underserved populations [4].

Our findings also reinforce national observations that self‐referral and primary care referral remain the central routes into behavioral WMS [34, 37], with secondary care playing a more limited role. While self‐referral routes can facilitate efficient access to services, an overreliance on them may inadvertently exclude those most in need of weight management support. For example, self‐referral requires patients to be aware of available services, motivated to navigate referral processes, and in some cases, digitally literate enough to complete online self‐referral forms. These skills may be limited among individuals from socioeconomically disadvantaged backgrounds, where low levels of digital literacy are common [38]. Similarly, primary care referrals depend on patients engaging with their GP or practice team and on HCPs recognizing patient eligibility and actively initiating their referral to appropriate services [39, 40]. This could disadvantage patients who infrequently access primary care, experience language or cultural barriers, or who have complex comorbidities that can overshadow overweight or obesity as a priority during consultations [41]. It is well documented that individuals from ethnic minority backgrounds are disproportionately unaware of publicly funded WMS, which are often seen to be poorly advertised [42].

The regional funding landscape was found to be similarly layered, with commissioning being primarily managed by Local Authorities and NHS England, while commercial and VCSE programmes required direct payment from the individual. It is notable that these patient self‐funded services were only moderately represented in the ecosystem maps (*n* = 4 of 11, 36.4%), particularly as they may be prohibitively costly for individuals from socioeconomically disadvantaged backgrounds or from ethnic minority groups, who often report travel and membership fees as additional barriers to participation [42]. This is particularly concerning given that high‐risk populations who stand to benefit most from behavioral WMS are also less likely to enroll in, or remain engaged with, these programmes [14, 43]. This finding reflects wider concerns that mixed funding arrangements may exclude those most likely to benefit from weight management support, which may in turn undermine equity goals embedded in national obesity strategies [44]. For example, the 2021/2022 Adult Weight Management Services Grant called for WMS provision to be prioritized for populations at higher risk of obesity, including those living in deprived areas, from minority ethnic communities, or those with mental or physical health conditions, in order to reduce disparities in obesity and obesity‐related health outcomes across the socioeconomic gradient [45]. To support accessibility and fairness, the future design and delivery of behavioral WMS should, where feasible, incorporate the views and preferences of underrepresented high‐risk groups in shaping how behavioral WMS are designed to reach them and meet their needs [46]. Practical considerations should nevertheless be acknowledged, as no single service can fully accommodate the diversity of needs across all individuals and communities [47].

By mapping the current regional behavioral WMS landscape, the ecosystem maps provide insight into key services, pathways, and inter‐stakeholder relationships, supporting the implementation of eSignposting to behavioral WMS as an adjunctive intervention that complements existing provision. For example, the maps make clear that regional WMS access is largely contingent on patients actively self‐enrolling into services or primary care practitioners initiating referral. eSignposting offers an opportunity to streamline access by circumventing potential bottlenecks. For example, automated text messages delivered via primary care practices rather than through secondary care settings, which play a more limited role in referral pathways, could substantially increase the reach and engagement of behavioral WMSs. However, an overreliance on existing referral pathways also carries the risk of reinforcing existing inequities if traditionally underserved groups are not specifically considered [44]. By leveraging electronic health record data on sociocultural and socioeconomic indicators, eSignposting can proactively identify high‐risk or underrepresented groups and direct them toward appropriate services, thereby bypassing barriers inherent in self‐referral systems and commercial self‐pay options. This targeted data‐driven approach can not only enhance engagement among underrepresented populations but also help balance demand across the system, supporting both equity and sustainability objectives.

The inter‐stakeholder relationships identified in the ecosystem maps may help to anticipate unintended system‐level consequences of eSignposting and allow for appropriate adjustments to the approach. For example, increased referral volumes resulting from eSignposting could strain Local Authority‐commissioned services, potentially increasing waiting lists or diluting program intensity and effectiveness, while leaving more accessible remote options underutilized. While this could be an indication for electronically signposting patients to services that offer remote delivery, funneling patients toward digital programmes could equally threaten the sustainability of smaller community‐based initiatives that may be better placed to address local health inequalities [48]. For instance, patients who receive automated text messages that electronically signpost them to a behavioral WMS may preferentially engage with services that offer simple online enrollment, as in the cases of commercial services such as Slimming World and WW. In contrast, Local Authority‐commissioned programmes that require patients to submit a self‐referral before undergoing manual follow‐up and administrative processing by service staff may see lower uptake, despite potentially being better suited to meet the needs of underserved populations [48].

Further risks exist around the digital divide, as populations with limited digital literacy or remote connectivity could be systematically excluded from receiving electronic text messages signposting them to appropriate WMS. While the vast majority of adults in the UK (more than 90% [49] own a mobile phone that enables them to receive standard text messages), disparities remain in the rates of phone ownership, internet access, and digital skills and confidence in using technology, particularly among socioeconomically disadvantaged groups [50]. In addition, reducing reliance on clinician‐facilitated referrals could unintentionally diminish opportunities to address weight management holistically during clinical consultations, where supportive, non‐judgmental conversations between patient and provider have been found to improve patient satisfaction with weight management support [51]. These considerations highlight the importance of careful design and ongoing monitoring of eSignposting to ensure that it strengthens rather than disadvantages the broader behavioral WMS ecosystem.

This study is the first to use ecosystem mapping to characterize a behavioral WMS ecosystem in a UK region. Input was gathered from multiple stakeholders involved in the delivery and commissioning of, and patient referrals to behavioral WMS to ensure that the developed ecosystem maps service inventory accurately and reflected local practice. Interview findings, combined with extensive online WMS database searches and stakeholder validation, further enhanced confidence in the completeness and reliability of the outputs.

There are also limitations to this study. The ecosystem maps focused on three counties in East England, resulting in a modest sample size of professional stakeholders involved in the regional behavioral WMS ecosystem (*n* = 11) and limiting generalizability of the findings to other regions. Obesity care pathways may operate differently in different regions and will require their own ecosystem mapping exercises to suitably inform the implementation of innovations such as eSignposting. In addition, recruitment via professional networks and snowball sampling may have introduced selection bias toward more well‐known or visible participants. Although the ecosystem maps were carefully validated, this could mean that less visible or newer services may have been overlooked. Furthermore, while all invited participants responded to the initial interview request, the response rate to subsequent requests for feedback on the maps and service inventory was 46%, potentially limiting the completeness and accuracy of the final outputs. Additionally, this study focused on the behavioral (Tier 2) WMS ecosystem and did not survey universal (Tier 1) interventions, such as the NHS Weight Loss Plan app, or specialist (Tier 3) pharmacological treatments, such as tirzepatide. These interventions represent distinct, and in the case of pharmacological treatments, rapidly expanding [52] components of the national obesity care pathway and warrant separate investigation. It is worth noting that the ecosystem maps represent a snapshot in time of the particular service landscape and will require periodic updating to reflect changes in service provision, incentives for screening and referral and commissioning arrangements. Nonetheless, they provide a valuable foundational resource to guide the implementation of eSignposting and can be readily revised as the landscape evolves.

This study identified the components of the behavioral WMS ecosystem and collated them into a comprehensive visual representation to support the effective and equitable implementation of eSignposting. In addition to providing a repository of services available for patient referral, the ecosystem maps outlined the complex interconnections among key stakeholders, illustrating how introducing eSignposting in one area could generate cascading effects across the ecosystem. Findings suggest that eSignposting could enhance access and equity by proactively connecting high‐risk or underrepresented patients to appropriate services. However, it should be thoughtfully implemented with attention to the persistent health inequities that characterize the weight management landscape, to avoid unintended consequences such as placing strain on local services or digitally excluding those most in need. More broadly, this approach demonstrates how visualizing complex health service ecosystems can guide the equitable design and implementation of adjunctive interventions, offering an approach that can be applied to other health service innovations.

## Author Contributions

Natalie An Qi Tham and Zarnie Khadjesari conceptualized the study. Natalie An Qi Tham conducted the interviews, analyzed the data, developed the ecosystem maps and service inventory, and drafted the manuscript. All authors have read and approved the final manuscript.

## Funding

The work was funded by Cancer Research UK (Grant RCCCEA‐Nov23/100002).

## Conflicts of Interest

H. M. P. is a British Obesity and Metabolic Surgery Society council member and an advisory panel member for the UK Coalition for People Living with Obesity. She has received honoraria for obesity related educational events and clinical pathway development consultancy from Johnson & Johnson, Novo Nordisk, Boston Scientific and Radcliffe Group. H. M. P. was a member of the NICE obesity clinical guidelines NG246 committee and NICE obesity quality standards for obesity QS212 committee. All other authors declare no conflicts of interest.

## Data Availability

All the data generated or analysed during this study are included in this article. Further inquiries can be directed to the corresponding author.
